# SquiggleNet: real-time, direct classification of nanopore signals

**DOI:** 10.1186/s13059-021-02511-y

**Published:** 2021-10-27

**Authors:** Yuwei Bao, Jack Wadden, John R. Erb-Downward, Piyush Ranjan, Weichen Zhou, Torrin L. McDonald, Ryan E. Mills, Alan P. Boyle, Robert P. Dickson, David Blaauw, Joshua D. Welch

**Affiliations:** 1 Department of Computer Science and Engineering, University of Michigan, Ann Arbor, 48109 MI USA; 2 Department of Electrical and Computer Engineering, University of Michigan, Ann Arbor, 48109 MI USA; 3 Division of Pulmonary and Critical Care Medicine, Department of Internal Medicine, University of Michigan Medical School, Ann Arbor, 48109 MI USA; 4 Department of Computational Medicine and Bioinformatics, University of Michigan, Ann Arbor, 48109 MI USA; 5 Department of Human Genetics, University of Michigan Medical, Ann Arbor, 48109 MI USA; 6 Department of Microbiology and Immunology, University of Michigan Medical School, Ann Arbor, 48109 MI USA; 7 Michigan Center for Integrative Research in Critical Care, Ann Arbor, 48109 MI USA

**Keywords:** Deep learning, Read-until, Oxford Nanopore, Raw signal, Real-time

## Abstract

We present SquiggleNet, the first deep-learning model that can classify nanopore reads directly from their electrical signals. SquiggleNet operates faster than DNA passes through the pore, allowing real-time classification and read ejection. Using 1 s of sequencing data, the classifier achieves significantly higher accuracy than base calling followed by sequence alignment. Our approach is also faster and requires an order of magnitude less memory than alignment-based approaches. SquiggleNet distinguished human from bacterial DNA with over 90% accuracy, generalized to unseen bacterial species in a human respiratory meta genome sample, and accurately classified sequences containing human long interspersed repeat elements.

**Supplementary Information:**

The online version contains supplementary material available at (10.1186/s13059-021-02511-y).

## Background

Oxford Nanopore sequencers, such as MinION or PromethION, determine the nucleotide sequence of a DNA or RNA molecule by measuring changes in electrical current (called “squiggles”) as the molecule translocates through a protein nanopore. This approach is fundamentally different from the widely-used Illumina platform and provides several benefits: the MinION is small, fast, and portable, making it ideal for rapid diagnostics and field work. Because it does not rely upon synchronized nucleotide addition (the heart of the Illumina sequencing-by-synthesis technology), MinION also produces much longer reads. To our knowledge, the longest published MinION read is around 2 Mbp [1], though even longer reads have been reported anecdotally. The changes in electrical current induced by a DNA or RNA molecule depend on the specific chemical properties of the nucleotides, including secondary structure interactions and epigenomic modifications such as methylation. Additionally, the nanopore sequencer can stream the squiggle data to a computer in real time.

The nanopore sequencer can also eject a partially sequenced molecule, a capability referred to as “Read-Until”. In principle, this enables targeted sequencing without the need for biochemical enrichment. The Read-Until capability allows selective sequencing of molecules by reversing the voltage across individually selected nanopores, ejecting the unwanted molecules. The unoccupied nanopores can then sequence different molecules of interest.

Such computational enrichment of target sequences holds great promise for clinical diagnostics and field research, but realizing this potential requires fast and accurate approaches for identifying molecules of interest. For example, identifying pathogenic DNA in a patient lung fluid sample requires bypassing human DNA—which often represents > 99*%* of the sequences—to find the pathogen sequences. Biochemical methods for target sequence enrichment, such as PCR [2–5], hybrid capture [6], or CRISPR/Cas9 enrichment [7, 8] require much more time, expertise, and equipment. In contrast, a computational approach to enriching target sequences provides clear savings of time, labor, and cost.

Previous computational approaches for this problem include (a) perform standard base calling followed by sequence alignment as in [9] and (b) perform rough base calling to identify and align *k*-mers [10]. The first approach requires significant computing resources—such as a graphics processing unit (GPU) and a large genome index database for the sequence aligner. The second approach also requires a large genome index and multiple CPU cores and can map only non-repetitive references smaller than ∼ 100 Mbp. Both approaches are based on sequence alignment and thus are limited by sequencing errors, their reliance on genome indexes, and their inability to capture non-sequence information such as DNA methylation.

To address these limitations, we developed SquiggleNet, the first deep-learning-based approach for classifying DNA sequences directly from electrical signals. SquiggleNet is fast, accurate, memory-efficient, and robust to unknown species. It requires only 3000 signals—less than the amount of data generated in one second of sequencing—to classify the species of a DNA molecule with over 90% accuracy, significantly higher than the best alignment-based methods. The model requires only 304 KB of RAM and no external reference database. SquiggleNet is faster than or on par with the competitors and can run in real time on a single core of a standard laptop. When tested on a human respiratory metagenome sample with a majority of unseen species, our approach achieves > 90*%* overall accuracy.

## Results

### SquiggleNet: a convolutional neural network for classifying nanopore signals

**Fig. 1:**
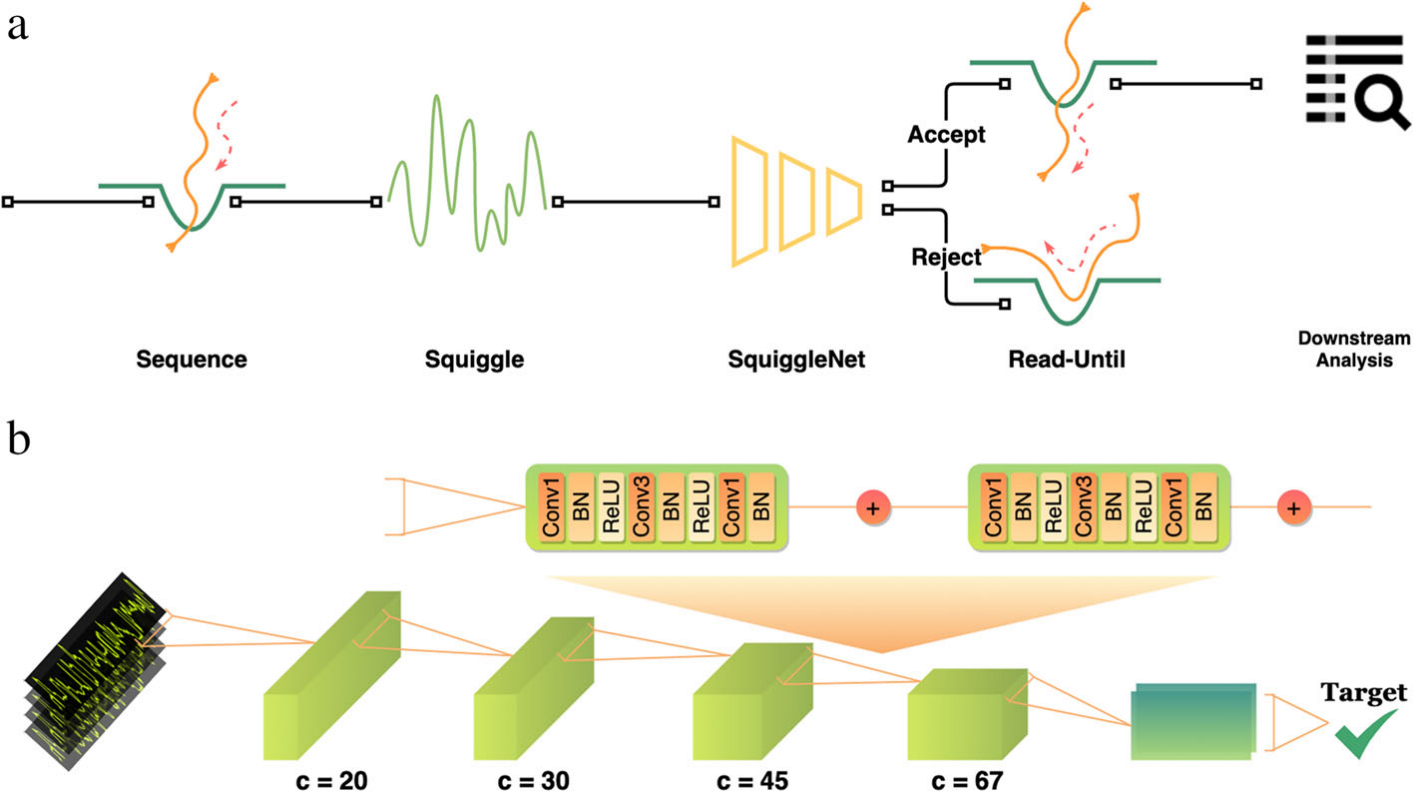
Read-until pipeline overview. **a** A DNA molecule translocates through a nanopore, generating electric signals (squiggles). SquiggleNet rapidly classifies the molecule to determine whether it is a sequence of interest. If the molecule is accepted by the classifier, it is sequenced to full length. Otherwise, the molecule is ejected from the pore, freeing the pore to sequence another molecule. **b** SquiggleNet employs 1D-ResNet-styled bottleneck blocks with increasing numbers of filters. Average pooling and a final fully connected layer are performed after the last convolutional block

SquiggleNet is a deep neural network that classifies molecules of interest based on statistical patterns in nanopore conductivity, which are often hard for humans to identify by eye, automatically extracted from the input data. The overall workflow for using SquiggleNet to enrich sequences of interest is shown in Fig. 1a. The network is first trained to recognize certain classes of sequences, such as human vs. bacterial DNA, using labeled examples. Then, as the nanopore sequencer generates raw electrical signals from a new and unseen sample, SquiggleNet rapidly classifies each molecule to determine whether it is a sequence of interest. Molecules not of interest are ejected from the nanopore, freeing the pore to sequence a different molecule. In contrast, targeted molecules are sequenced to full length and used for downstream analysis.

**Fig. 2:**
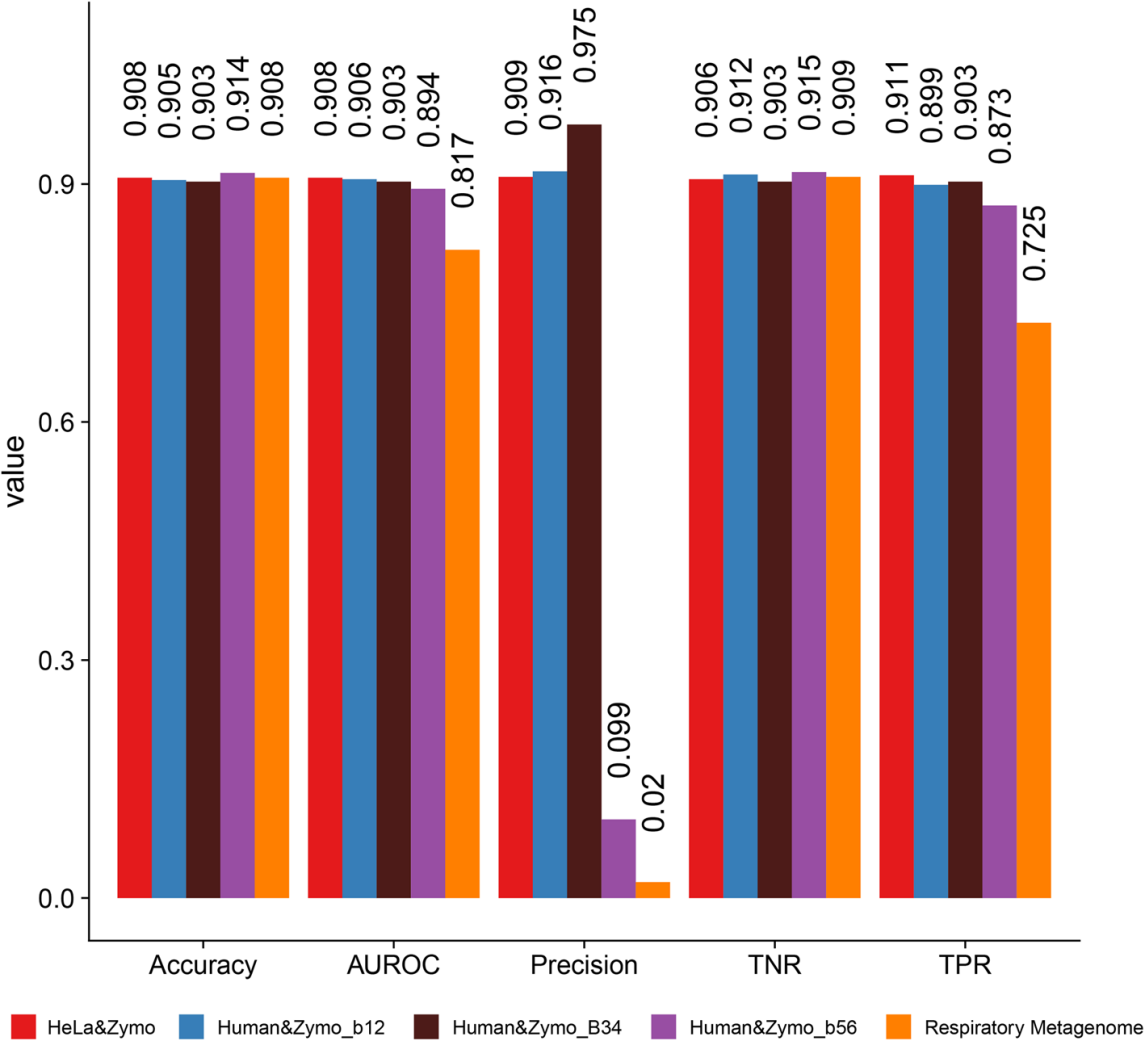
Overall performance across five test datasets: accuracy, true positive rate (TPR, RECALL), true negative rate (TNR), precision, and the AUROC score of the model trained on the HeLa&Zymo training set, and tested on five test sets with bacterial sequences as the target

SquiggleNet (Fig. 2b) employs a convolutional architecture, using residual blocks modified from ResNet [11] to perform one-dimensional (time-domain) convolution over squiggles. The architecture consists of four blocks with increasing numbers of channels; each block includes two 1D-ResNet Bottleneck units. Mean pooling followed by a fully-connected layer with softmax activation allows SquiggleNet to classify sequences based on the convolutional filters in the last ResNet block. The final output is a conditional probability on the sequence labels, which is then used to make the final class prediction. We experimented with several other approaches, including a recurrent neural network (RNN) with long short-term memory (LSTM) blocks; gated recurrent units (GRUs); other types of convolutional blocks; a combination of RNN and convolution; different convolutional window sizes; and differing model hyperparameters. However, we found that approaches based on convolution outperformed models using LSTM blocks, suggesting that local features are sufficient for this problem, and long-range time-dependent relationships do not add much information. Convolutional architectures without LSTM blocks are also faster to train. Our final architecture gave the best classification accuracy of any approach we tried and could not be made significantly smaller without sacrificing performance. Additional details about the model architecture and hyperparameter choices can be found in the Method section and Additional file 1.

### SquiggleNet accurately classifies species directly from squiggles

To test the performance of SquiggleNet, we generated four experimental datasets containing a mixture of human and bacterial DNA. The first dataset, HeLa&Zymo, contains 8 bacterial species from the Zymo mixture [12] and HeLa cells. The species labels were obtained through Minimap2 [13] alignment. The other three datasets (Human&Zymo_b12, Human&Zymo_b34, and Human&Zymo_b56) contain a mixture of human GM12878 DNA and DNA from Zymo High Molecular Weight mixture with 7 bacterial species [14]. To avoid systematic error from the alignment algorithms, we obtained reliable ground-truth species labels for these three datasets by attaching a nucleotide sequence barcode to each DNA molecule indicating whether the molecule is from human or bacteria. Note that our direct biochemical labeling strategy allows us to independently assess the accuracy of species determination from base calling followed by read alignment; this is important for our application, since we expect that SquiggleNet may be able to outperform purely sequence-based approaches by leveraging other information from the electrical signals. Further dataset details can be found in the Method section and Additional file 1.

We trained SquiggleNet using more than two million reads from the first dataset (HeLa&Zymo), which contains equal proportions of HeLa and bacterial sequences. We used 3000 signals from each read, the equivalent of about 300 nucleotides. We discarded the first 1500 signals of each read (an overestimation of the adapter length), to remove potential pore noise and adapter sequences, which could confound training. Thus SquiggleNet requires a total of 4500 signals, which is equivalent to about 1 second of sequencing time. (The exact time and number of nucleotides depends on the translocation speed, which varies per pore and molecule over the course of the sequencing experiment.) However, using this exact amount of signal is not crucial; we verified that using fewer signals did not significantly change the results (see below). Our best-performing model was trained on the HeLa&Zymo dataset, which contains the largest number of sequenced reads. This dataset also lacks species-specific barcodes, and we were careful to remove the sequencing adapters and species barcodes before extracting the 3000 signals used for classification (Methods). Thus, there is no way that the classifier could “cheat” by using the barcodes to classify the species. When we instead trained the model on the Human&Zymo datasets and tested on HeLa&Zymo, the model accuracy was nearly identical but slightly lower, possibly due to the smaller number of training samples (see Additional file 1: Figure S1).

Overall, the model classifies each molecule as bacterial or human with over 90% accuracy across different test datasets using only 3000 signals per read (see Fig. 2). The classifier generalizes well to different lab preparations, flow cells and proportions of species. For the first three datasets (HeLa&Zymo, Human&Zymo_b12, Human&Zymo_b34), the true positive rates (TPR, also Recall) and the true negative rates (TNR) are all above or around 90%. The precision and AUROC scores are all about 90% as well. Even for samples with significantly more human than bacterial DNA (Human&Zymo_b56 and Respiratory Metagenome), the accuracy and recall both remain high.

We used the method of integrated gradients (IG) [15] to investigate the features influencing SquiggleNet’s classification decisions. The IG method computes the amount of gradient change for each corresponding input, and by doing so, offers interpretation on which part of the input contributes the most to the model’s decision. Inspecting these IG results (Additional file 1: Figure S2) shows that SquiggleNet predictions are most strongly influenced by positions where the signal changes direction, changes by a large amount, and/or changes from one nucleotide to another. This suggests that SquiggleNet has learned filters related to the nucleotide composition of the signal and uses the results to make classification decisions. Further details are contained in the Additional file 1.

To demonstrate that the results are robust to the amount of signal removed from the beginning of the read at test time, we also tested our pre-trained model on the Human&Zymo_b34 dataset with only the first 1000 signals per read removed. We chose the number 1000 because this is a closer estimation of the adapter length [16], and at test time, we would like to make the decision as soon as possible to enable real-time read selection. When testing the model on sequences with the first 1000 signals removed, the results were nearly identical to those obtained from conservatively removing 1500 signals: 89.35% accuracy, 90% true positive rate, and 86.9% true negative rate. Thus it appears that, as long as the initial pore noise, adaptors, and barcodes are removed from the training sequences, the model is able to make an accurate and fast decision at test time. This robustness also allows flexibility if, for example, different sequencing datasets use sequencing adapters of different lengths.

Remarkably, we find that SquiggleNet achieves significantly higher accuracy from 3000 signals than base calling followed by sequence alignment using the same amount of signal. This result gives crucial context for interpreting the accuracy of our model and suggests that the convolutional filters may detect some non-sequence features that help with species classification, such as chemical modification of nucleotides by methylation. Indeed, we found that the bacterial and human DNA sequences in our dataset show significant methylation differences, with significantly more methylated cytosines in human sequences and significantly more methylated adenines in bacterial sequences (see Additional file 1: Table S2 for details).

For the Human&Zymob_56 dataset, the target to non-target sequence ratio is 1 to 99. The overall accuracy, TNR, and AUROC score are around 90%. The TPR (Recall) is closely following, above 87%. The precision, however, is about 1/10 of the other cases. This is due to the extremely low concentration of the target sequences (Zymo bacterial species), and the precision calculation is diluted by the overwhelming number of false positive reads. Nevertheless, this is acceptable, since we want to preserve as many targeted reads as possible (high recall) due to the low target read concentration. All true positives and false positives will be sequenced to full length, and thus can be further processed in the downstream analysis. Considering that the Human&Zymo_b56 dataset has 99 × more non-targeted reads than targeted, whereas only ∼ 10× more reads were falsely identified as positive compared to the HeLa&Zymo dataset, this model demonstrated strong ability to filter out non-targeted reads, and has high potential to improve throughput (see below). Overall, the model that was trained on only the HeLa&Zymo dataset yields high performance across different testing datasets, highlighting the robustness of the model.

**Fig. 3:**
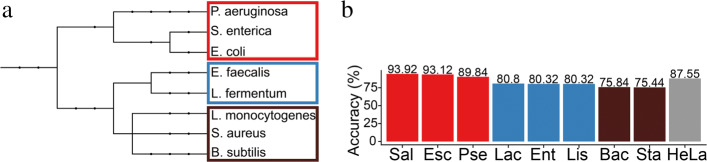
Taxonomy tree and accuracy per species. Taxonomy tree for the eight species in our dataset grouped in color and their corresponding accuracy breakdown per species. The accuracy for distinguishing bacterial sequences from human was highest for the red branch, intermediate for the blue group, and lowest for the brown group

Interestingly, SquiggleNet performance varies systematically across bacterial taxa. The network classifies human vs. bacterial DNA with 90% accuracy, but some bacterial species are easier to distinguish from human sequences than others. The eight bacterial species in the Zymo mixture are related according to the taxonomy tree shown in Fig. 3. The top three species—*Pseudomonas aeruginosa* (Pse), *Salmonella enterica* (Sal), and *Escherichia coli* (Esc)—are gram-negative bacteria and are most easy to identify, while the bottom five species are gram-positive bacteria and are harder to distinguish from human DNA. It is not clear what specific features of the gram-negative bacteria make them easier to identify, but this behavior may be related to species differences in GC-content or the amount of methylation.

### SquiggleNet identifies species not seen during training

In real-world applications, samples may contain species whose genomes are not in the training samples. We thus investigated whether the model can identify unseen species. To do this, we performed a leave-one-out analysis, removing each of the bacterial species separately during training, then putting it back during testing to challenge SquiggleNet’s generalization ability. For the held-out species comparisons, we used 400k and 20k reads from the HeLa&Zymo dataset for training and testing, respectively.

During each training run, we removed one of the eight Zymo bacterial species from the training dataset. We then compared the test accuracy from the classifier trained on seven bacterial species plus human with the performance of the same model on two different testing sets containing the eighth held-out species. The dataset we call Test-Uniform/HeLa includes all eight species (including the one held out during training), evenly distributed, and balanced to contain equal numbers of HeLa and bacterial molecules. The dataset we refer to as Test-One/HeLa includes only the single held-out species and HeLa, in equal proportions.

**Fig. 4:**
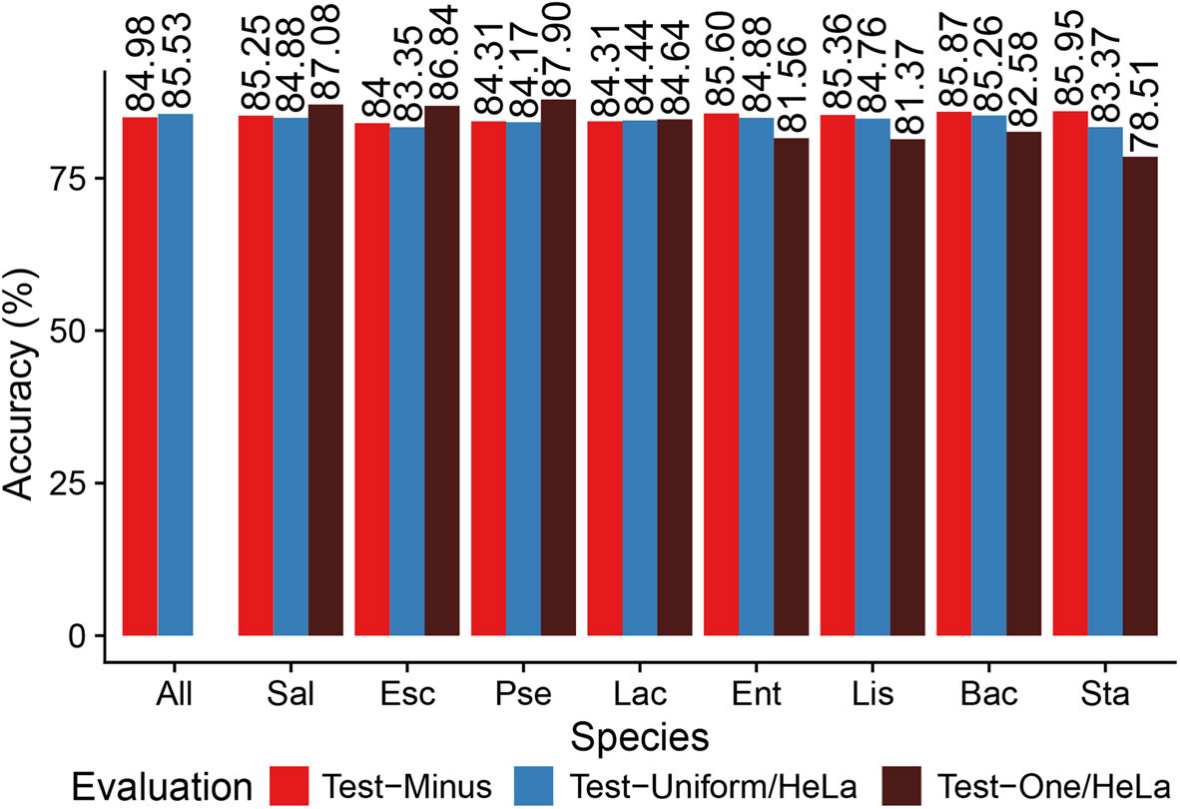
Performance of SquiggleNet on unseen species. Each column (except “All”) is a model trained on a Zymo/HeLa 1:1 mix without the held-out species. For each species, the red bar shows the test accuracy on all species minus the held-out species; this number provides a baseline against which to compare performance on the held-out species. Blue bars show the accuracy of each trained model on Test-Uniform/HeLa, a test set with all eight Zymo bacterial species included and HeLa in a 1:1 ratio. Brown bars show the accuracy of each model on Test-One/HeLa, a test set with only the single unseen species and HeLa in a 1:1 ratio

The unknown species identification results can be found in Fig. 4. The red bars are the test accuracy results without held-out species. The left-most column is the performance of a training run with all 8 bacterial species as a reference for cross-testing run performance comparison.

Across different runs, the test accuracies, not including held-out species, are around 84–86%. For each Test-Uniform/HeLa experiment, accuracy of classifying the held-out species was ∼ 83–85%, only about 1% lower compared to when the species was seen during training. This shows that the model was able to accurately identify sequences from bacterial species that were not seen during training. For the Test-One/HeLa experiment, the test performance is more influenced by the taxonomic position of the held-out species. Since the testing datasets only include human DNA and the one species that was held out, we expected performance to drop even more than the previous Test-Uniform/HeLa experiment. However, the test accuracies of the first three gram-negative bacterial species, *Pseudomonas aeruginosa* (Pse), *Salmonella enterica* (Sal), and *Escherichia coli* (Esc) actually increased by ∼ 1–4% compared to their validation accuracies. The remaining four gram-positive species had a minor performance increase or drop within 4%. *Staphylococcus aureus* had the largest performance drop among all, but the accuracy was still above 75%.

In summary, these two sets of experiments show that even when one species was not seen during training time, SquiggleNet was still able to identify it with high confidence.

### SquiggleNet identifies bacterial DNA in a human respiratory metagenome sample

**Fig. 5:**
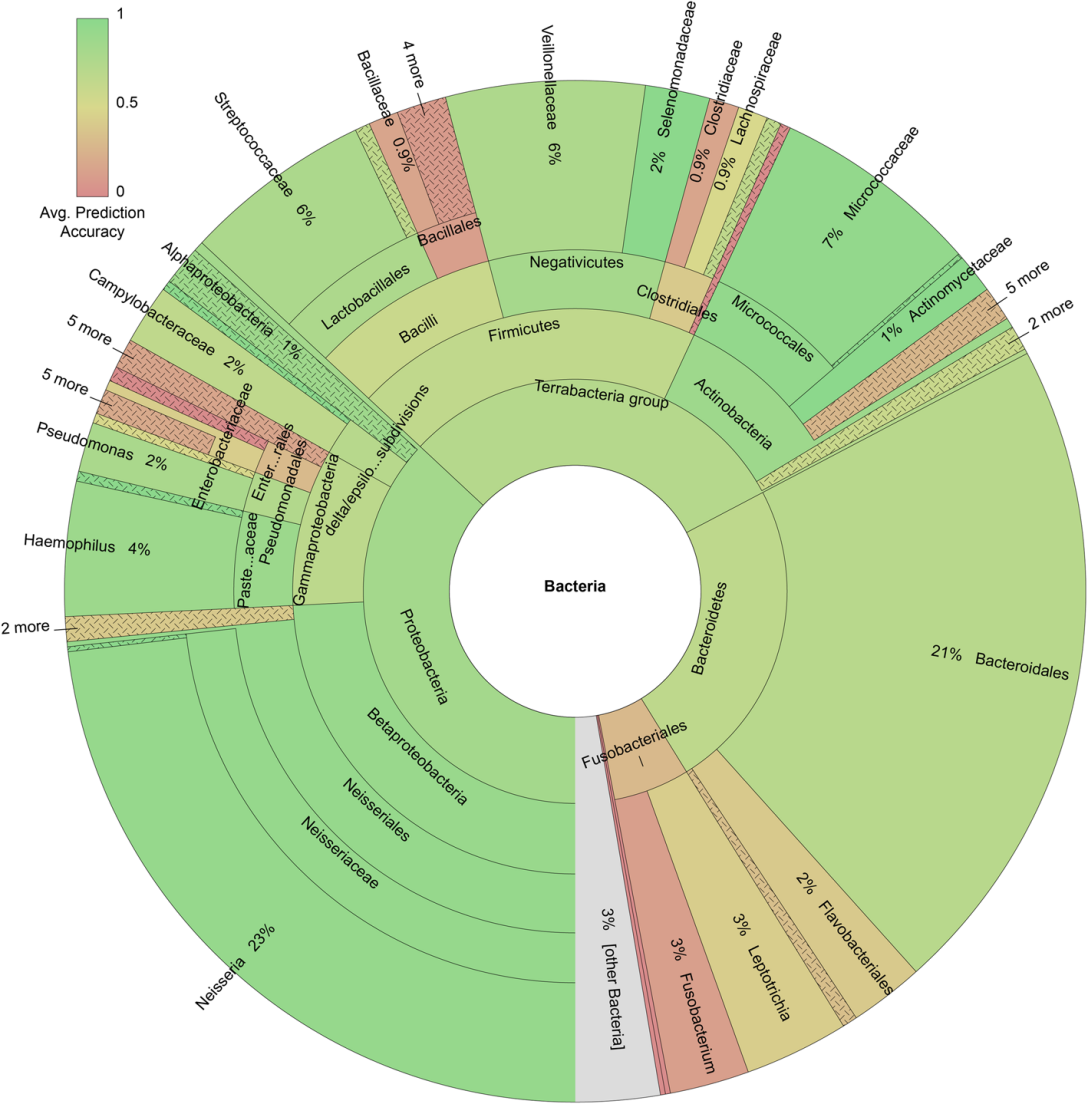
SquiggleNet accuracy by species in human respiratory metagenome sample[19]. Some of the dominant bacterial groups include *Neisseria* (23%), *Bacteriodales* (21%), and *Firmicutes* (20%). Less than 3% of the bacterial species overlap with the training dataset

To further test the generalizability and practicality of SquiggleNet, we tested the best performing model (trained on the Hela&Zymo dataset) on a dataset collected from several clinical human samples. We collected the data following the procedures in [17]. The ground-truth labels were obtained using our previously published read alignment pipeline[18]. The dataset includes 324,526 human reads and 341 bacteria and other (less than 0.6%), a human:bacterial ratio of 951:1. Some of the dominant bacteria groups include *Prevotella* (29%), *Neisseria* (20%), and *Rothia* (11%). However, less than 3% of the bacterial species overlap with the training dataset. The full taxonomic composition can be found in Fig. 5 and [17].

Even though the model was trained on dataset Hela&Zymo, it achieved 90.8% overall accuracy in the Respiratory Metagenome dataset, 72.5% true positive rate, and 90.9% true negative rate (Fig. 2). The AUROC score is 0.817. The precision is about 1/5 of that in dataset Human&Zymo_b56. As with the unbalanced Human&Zymo dataset, the precision is diluted by the extremely low concentration of bacteria, but the model still achieves high recall—which is critical to retrieve all the bacterial reads for downstream analysis.

The Zymo community of the dataset on which the model was trained has very little overlap (< 3*%*) with the bacterial species found in the Respiratory Metagenome dataset. The genome information in this testing dataset was mostly unseen and unknown for the trained model. However, it still achieved a true positive rate of 72.5%. This shows that SquiggleNet is able to extract common bacterial genome features and distinguish them from the human genome sequencing raw signals. The generalizability of SquiggleNet significantly increases the potential applications of our method. As shown in Fig. 5, different species were classified with different accuracy. The model is therefore, expected to be even more accurate if it can be fine-tuned in a dataset with closer range of species.

### SquiggleNet is more accurate and efficient than previous approaches

We next compared the performance and efficiency of SquiggleNet against the current state-of-the-art methods: Guppy+Minimap2 and UNCALLED. This experiment was conducted on dataset Human&Zymo_b34 with 1:4 Human and Zymo mix. All the analysis was done on a single-usage Intel(R) Xeon(R) CPU E5-2697 v3 @ 2.60GHz machine with a single TITAN Xp GPU.

**Fig. 6:**
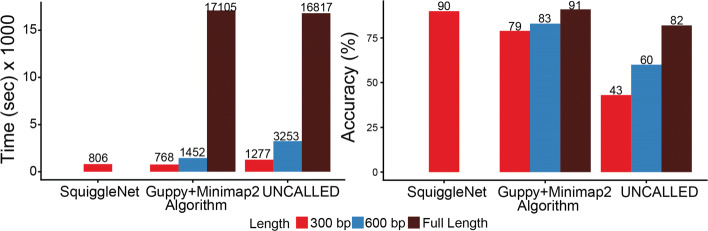
Processing time and accuracy comparison. The processing time of SquiggleNet with 300 bp of input is among the lowest, and yet the accuracy is the highest among the three methods. For the other two alignment-based methods, with longer input length, the processing time grows drastically, whereas the accuracy gain is limited

We benchmarked the running time required to classify 712,000 reads (178 fast5 files with 4000 sequences each and 3000 signals per read, adapters and barcodes removed). SquiggleNet took 806.74 s (13 min 27 s) to finish processing all on GPU (Fig. 6). When tested on a 3.5-GHz Dual-Core Intel Core i7 Macbook Pro, SquiggleNet finished processing all the files in 2630.58 s (43 min 51 s). With the Guppy+Minimap2 method [9], sequences were base called using Guppy [20] with 4 callers and 4 runners/GPU, and we used 32 threads for sequence alignment with Minimap2 [13]. It took 742.602 s (12 min 23 s) for Guppy to finish base calling 3000 signals and another 25.673 s for Minimap2 to finish the alignment, about the same amount of time as SquiggleNet. The accuracy of Guppy+Minimap2, however, was 79%, more than 10% lower than SquiggleNet. Using the full length of the input sequence increased the accuracy of Guppy+Minimap2 to 91%, but the processing time increased dramatically. With UNCALLED, 32 threads were used to process 3000 signals, 6000 signals, and full-length reads respectively. It took at least 1277.28 seconds (21 min 17 s) to finish the 3000 signals, but the accuracy was below 50%. With longer input length, the accuracy increased to 60% (82% for full-length), but the full length accuracy was still lower than SquiggleNet with 3000 signals. Meanwhile, the processing time grows drastically as well. SquiggleNet is, therefore, faster and more accurate than either Guppy+Minimap2 or UNCALLED.

Note that the accuracy values that we report take into account all reads, including those that Minimap2 could not align. In contrast, the UNCALLED paper reported that the method was able to recover 94% of the alignments identified by Minimap2, but this number does not take into account the reads that Minimap2 failed to align. Crucially, our datasets have molecular barcodes, allowing us to determine the true species even for reads that Minimap2 failed to align. Furthermore, the 94% accuracy reported by the UNCALLED paper is based on using the entire sequence, whereas we only used significantly less information (3000 signals) to classify the reads.

We also observed that over 90% of the SquiggleNet processing time on GPU and over 40% of the processing time on CPU is spent on loading data from the disk. The actual classification time for a batch of 500 reads is about 0.06 seconds on GPU and about 0.8 seconds on CPU (one thread). When streaming directly from the nanopore sequencer in real time, this loading time can be significantly reduced.

SquiggleNet also offers significant advantages in terms of space requirements (Table 1), requiring only 304 KB to store the model parameters. The run-time space usage is dominated by the storage required for each mini-batch of sequences, rather than the model parameters. Guppy, however, is a much larger deep-learning model, and the smallest pre-trained option available through Oxford Nanopore Community [20] is 5.5MB. On top of that, however, the Guppy+Minimap2 method also requires a customized database for Minimap2 reference. In this experiment, the human and Zymo reference database takes 3.2GB. UNCALLED is currently operational only on CPU. Similarly, it also takes a reference database to build a Burrows-Wheeler index, which is an extra 3.2 GB in this experiment. Therefore, SquiggleNet requires much less space than the other two methods.

**Table 1:** Method requirement comparison

	SquiggleNet	SquiggleNet	Guppy+Minimap2	UNCALLED
Equipment (*t* = thread)	GPU	CPU (*t* = 1)	GPU+CPU (*t* = 32)	CPU (*t* = 32)
Space requirement *↓*	**≤ GPU Max**	**≤ Mem Max**	≤ GPU Max + 3.2GB	≤ Mem Max + 3.2GB
Model size *↓*	**304 KB**	**304 KB**	5.5/40/116 MB	

### SquiggleNet identifies reads containing human long interspersed repeat elements

**Fig. 7:**
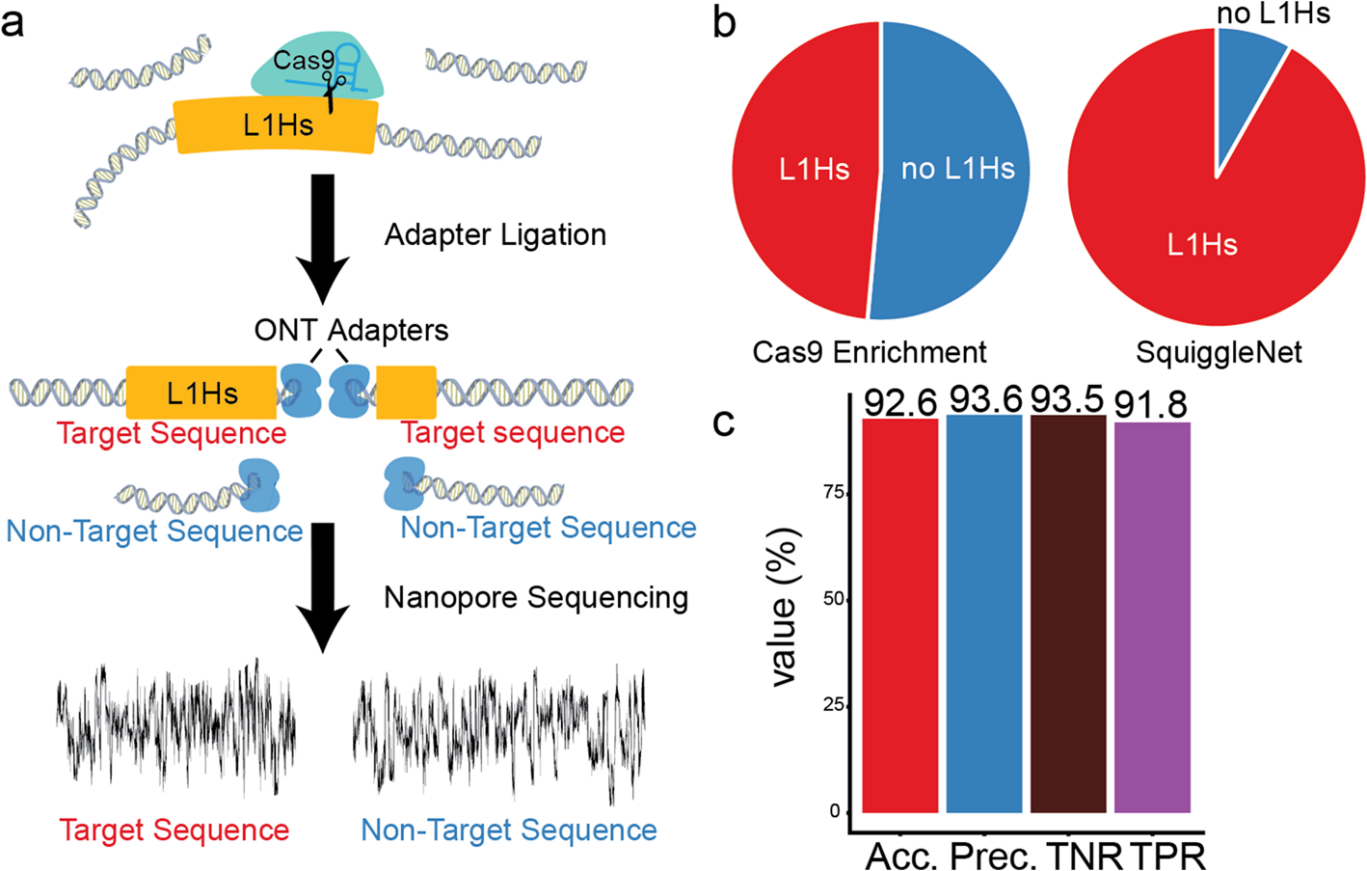
Identifying reads containing human long interspersed repeat elements. **a** Diagram of experimental strategy for enriching human mobile elements, including interspersed repeats. A guide RNA specific to each repeat class directs Cas9 to cut the DNA and ligate a sequencing adapter. However, adapters are also ligated to some sequences without repeat elements. Subsequent nanopore sequencing produces both target and non-target reads. **b** Pie charts of the proportion of L1Hs repeat elements from Cas9 enrichment only vs. SquiggleNet classification. **c** Classification metrics demonstrating SquiggleNet’s ability to distinguish reads with or without L1Hs repeats

Classifying species is useful for many applications, but distinguishing among different loci from the same species would significantly expand the settings in which SquiggleNet can be applied. To investigate whether SquiggleNet can be used to enrich loci of interest within a single species, we analyzed data from a recently published protocol [21] that enriches specific families of interspersed repeat elements using Cas9-directed adapter ligation (Fig. 7a). This experimental enrichment is effective, but still fewer than 50% of reads contain the repeats of interest [21]. As previously described [22], we used BLASTn [23] on the base-called sequence of each read to label the reads as target (repeat-containing) or non-target (no repeat). We focused specifically on a single class of interspersed repeats, human-specific long interspersed elements (L1Hs), which was the family most effectively enriched by the Cas9-directed ligation protocol. Using these labels, we trained SquiggleNet on a balanced dataset containing approximately 170,000 reads from each class. Note that this is about tenfold less training data than we used in the human vs. bacterial classification experiments above. As with the species classification experiments, we discarded the first 1500 signals from each read, then used the next 3000 for training or testing. Despite the smaller training dataset, we found that SquiggleNet was extremely effective at identifying reads containing L1Hs elements (Fig. 7b, c). Our model achieved more than 92% accuracy with a true positive rate above 93%. These results indicate that using SquiggleNet in a read-until setting to enrich long interspersed repeats would provide a significant benefit compared to Cas9 enrichment only.

### SquiggleNet improves throughput by enabling computationally targeted sequencing

To assess the potential improvement in sequencing throughput that SquiggleNet could provide, we developed a mathematical model to compare the total number of base pairs and total sequencing time needed to obtain a certain number of targeted sequences with and without SquiggleNet.

The detailed derivation of the model can be found in Additional file 1. The most influential hyper-parameters include average target sequence length $\bar {z}$, average non-target sequence length $\bar {h}$, and target sequence concentration *c*. Several other tunable hyper-parameters, including the waiting time to eject one molecule and begin sequencing another; the total number of active pores in a flow cell; the sequencing speed; and the total number of targeted sequences, did not significantly influence the predicted increase in throughput (see Additional file 1). We chose the values for these less influential parameters based on the empirical time requirements and accuracy of SquiggleNet and the sample means from the real sequencing data.

**Fig. 8:**
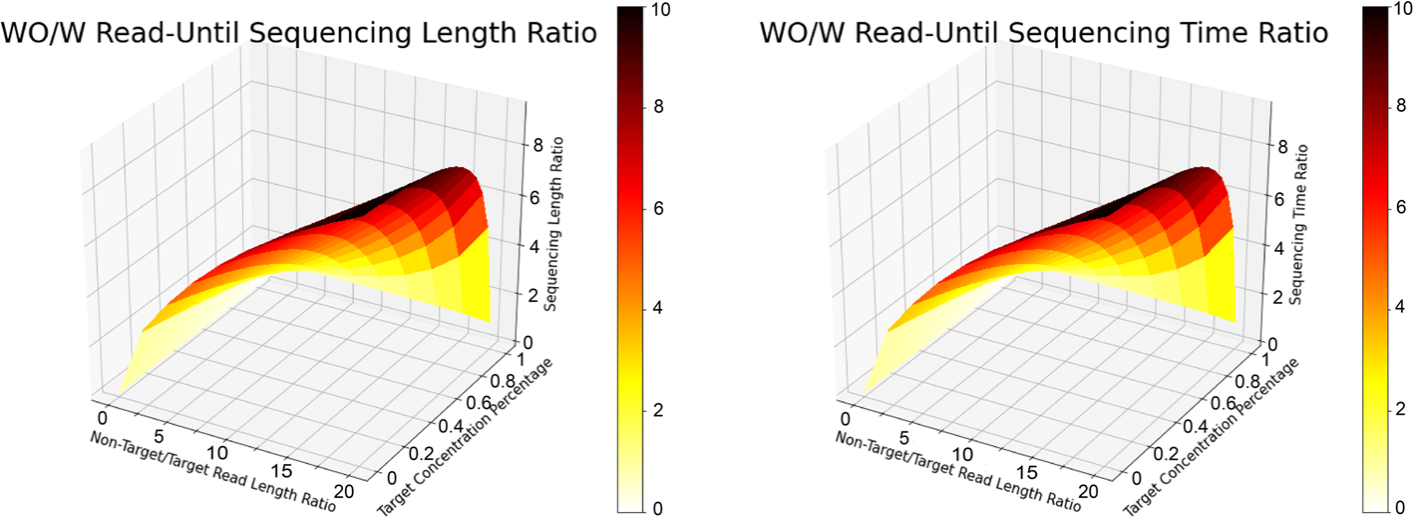
Throughput and sequencing time comparison without/with read-until. When the average non-target read length is about 20 times longer than the target read length, and sample contains over 90% non-target reads, a normal sequencing pipeline would have to sequence ∼ 10 times more base pairs (left) than Read-Until pipeline with SquiggleNet to achieve a fixed number of targeted reads. The ratio is about 10 for the required sequencing time as well (right)

In Fig. 8, we picked the average non-target/target sequence length ratio as one axis, and the target sequence concentration as the other axis to demonstrate the total number of base pairs (left) and total sequencing time (right) that a regular nanopore sequencing pipeline would require, compared to those of a pipeline with SquiggleNet, in order to obtain a fixed number of targeted reads. Based on the properties of our sequencing datasets, we set average sequencing speed to be 450 base pairs per second and the total number of active pores in a flow cell to be 500. We also used the following parameters based on SquiggleNet’s empirical performance: TPR = 0.9, TNR = 0.9, sequencing time = 1s, and classification decision time = 0.8 s (time required for SquiggleNet on CPU).

We show the predicted gains in throughput and sequencing time for a range of the most important hyperparameters (Fig. 8). When the average non-target read length is about 20 times longer than the target read length, and the sample contains over 90% non-target sequences, it would take a nanopore sequencing pipeline ∼ 10 times longer than Read-Until pipeline with SquiggleNet to achieve a fixed number of targeted reads. The regular nanopore sequencing pipeline would also have to sequence ∼ 10 times more base pairs than the Read-Until pipeline. Even if we set these parameters much more pessimistically, the model still predicts about a 5-fold gain in throughput and time. These numbers are also in the same ballpark as the 4.5 × enrichment reported in the UNCALLED paper [10], supporting the plausibility of our mathematical model. We therefore conclude that Read-Until with SquiggleNet holds great promise to improve target read throughput, saving sequencing time and resources.

## Discussion

The success of our approach suggests that the raw sequencing signals generated by nanopore sequencing contain rich information for identifying target sequences from background sequences. Such features could include different DNA modification patterns, codon frequencies, GC content, or even DNA shape or RNA secondary structure. Furthermore, because these features are primarily local in nature, only a small amount of sequencing signal is required. In contrast, approaches that rely on sequence information alone require much more signal (more sequencing time), are susceptible to base-calling errors, and do not leverage non-sequence information.

We also note that different reads go through the pores at different speeds. Future work could also include an event detector and a scaler into the classifier, which may further improve performance. Additionally, the squiggles from different MinION flow cells show systematic run-to-run differences. Thus, the data preprocessing and normalization procedures that we employed are crucial for generalizing across datasets.

We tested the capability of our model to enrich bacterial DNA in the presence of more abundant human DNA. Human and bacterial DNA are significantly different, which makes this classification task feasible. We also demonstrated that SquiggleNet can identify target sequences in other contexts, such as interspersed repeat elements within the human genome. This suggests that SquiggleNet could be used for targeted enrichment of other genomic loci, such as commonly mutated cancer genes or regions that are highly polymorphic across the human population. We also anticipate that SquiggleNet will be useful for distinguishing viral DNA or RNA sequences from host molecules within infected cells. Rapidly identifying targeted sequences could be helpful in numerous clinical settings, including cancer diagnosis, respiratory pathogen identification, and coronavirus testing.

## Methods

### Data collection

We generated five datasets using a MinION sequencer (Table 2) for this paper. The first four datasets used the standard Rapid Sequencing Kit (SQK-RAD004) protocol on a FLO-106D MinION Flow Cell. The HeLa&Zymo dataset used the ZymoBIOMICS Microbial Community DNA Standard, and datasets 2–4 used the ZymoBIOMICS HMW DNA Standard with different barcodes specified in Table 2. Details about the dataset composition can be found in Additional file 1. The Respiratory Metagenome data collection method can be found in [17].

**Table 2:** Datasets description

Name	Ratio	Train	Validation	Test	Note
HeLa&Zymo	1:1	2.4M	40k	54k	
Human&Zymo_b12	1:1	1.72M	40k	44k	Barcode 1 and 2
Human&Zymo_b34	1:4			224k	Barcode 3 and 4
Human&Zymo_b56	99:1			100k	Barcode 5 and 6
Respiratory Metagenome	951:1			324,867	Real patient samples

Because base calling and sequence alignment of noisy nanopore reads can result in systematic errors and is not a completely reliable source of ground truth, we used barcodes to label the sequences in the three Human&Zymo datasets as either bacteria or human before mixing them together. The labels obtained in this way thus represent reliable ground truth.

Each extracted signal read was normalized with fast5 scaling and offset. All reads were also normalized using *Z*-scored median absolute deviation. The extreme signal values with a modified *z*-score larger than 3.5 were replaced by the average of closest neighbors.

### Model architecture

SquiggleNet is a 1D-ResNet-based binary classifier (Fig. 1). The first layer of 1D-CNN is comparable to the first layer of Guppy[20], but with significantly fewer channels (20 instead of 512). After that, there are four layers of 1D-ResNet, and each layer includes two BottleNeck blocks. The number of channels for each layer increases by a factor of 1.5, and each BottleNeck block decreases the string size with a stride of 2. We perform average pooling after the final convolutional layer, followed by a fully connected layer. We also experimented with other architectures (see Additional file 1).

### Training and evaluation

Our best-performing model was trained on the HeLa&Zymo dataset with binary cross-entropy loss. The dataset was split into training, validation, and testing sets. The Human&Zymo_b12, Human&Zymo_b34, Human&Zymo_b56, and Respiratory Metagenome datasets were used to assemble testing sets for the best-performing model.

As a separate analysis, we also trained on the Human&Zymo datasets and tested on the HeLa&Zymo datasets. The performance of this model was nearly identical but slightly worse than the model trained on HeLa&Zymo (see Additional file 1 for details).

The Adam optimizer was used for over 6 epochs on each dataset, with a learning rate of 1e-3 and batch size of 1000. The model was initialized using Kaiming initialization in fan-out mode. Batch normalization was conducted within each Bottleneck block.

For the human interspersed repeat analysis, we used a balanced training dataset with equal numbers of reads that contained and did not contain an L1Hs element (about 170,000 reads each). We identified L1Hs elements using BLAST on the base-called reads with an *e*-value cutoff of 1×10^−5^, as previously described [21]. We trained for 3 epochs using the Adam optimizer. Evaluation metrics include overall accuracy, true positive rate (TPR, Recall), true negative rate (TNR), Precision, and area under receiver operating characteristic curve (AUROC) when running the model on different test datasets. Speed and memory comparisons were performed on the same Intel(R) Xeon(R) CPU E5-2697 v3 @ 2.60 GHz machine with a single TITAN Xp GPU.

## Supplementary Information

**Additional file 1** Supplement. Details on data collection, model parameters, hyperparameter tuning, details on the efficiency comparison experiment method, details on the theoretical throughput estimation model are included in the Supplement. Additional experiment results, including DNA methylation, model interpretation, model performance trained on a different dataset, model performance on different test dataset composition ratio and on odd/even chromosomes, are also presented in the Supplement [24–28].

**Additional file 2** Review history.

## Data Availability

We have uploaded our data to SRA (SRP296988) [29, 30]. The human metagenome dataset has been previously published in [17]. The Cas9 enrichment dataset is previously published and available on the SRA (BioProject accession PRJNA699027)[21]. The code is available at a DOI-assigning repository Zenodo (10.5281/zenodo.4728278) and under the MIT license in our Github repository [31]: https://github.com/welch-lab/SquiggleNet.
