# Supplementary material for: SquiggleNet: real-time, direct classification of nanopore signals
Source: Genome Biol. 2021 Oct 27;22:298. doi: 10.1186/s13059-021-02511-y (PMC8548853; doi:10.1186/s13059-021-02511-y)
Supplement: Supplementary file 1 — Additional file 1 Supplement. Details on data collection, model parameters, hyperparameter tuning, details on the efficiency comparison experiment method, details on the theoretical throughput estimation model are included in the Supplement. Additional experiment results, including DNA methylation, model interpretation, model performance trained on a different dataset, model performance on different test dataset composition ratio and on odd/even chromosomes, are also presented in the Supplement [24–28]. [file 13059_2021_2511_MOESM1_ESM.pdf]

Supplement

Data Collection

We generated five datasets using a MinION sequencer (Table 2) for this paper. The first four datasets used the standard Rapid Sequencing Kit (SQK-RAD004) protocol on FLO-106D MinION Flow Cell. The HeLa&Zymo dataset used the ZymoBIOMICS Microbial Community DNA Standard, and Datasets 2−4 used the ZymoBIOMICS HMW DNA Standard with different barcodes specified in Table 2. The Respiratory Metagenome data collection method is described in [17].

The theoretical composition of the ZymoBIOMICS Microbial Community DNA Standard [12] includes 8 types of bacteria with 12% each of: *Listeria monocytogenes* (Lis), *Pseudomonas aeruginosa* (Pse), *Bacillus subtilis* (Bac), *Escherichia coli* (Esc), *Salmonella enterica* (Sal), *Lactobacillus fermentum* (Lac), *Enterococcus faecalis* (Ent), *Staphylococcus aureus* (Sta); and 2 types of fungi (2% each of *Saccharomyces cerevisiae* and *Cryptococcus neoformans*). The theoretical composition of the ZymoBIOMICS HMW DNA Standard [14] includes 7 types of bacteria with 14% each of *Listeria monocytogenes*, *Pseudomonas aeruginosa*, *Bacillus subtilis*, *Escherichia coli*, *Salmonella enterica*, *Enterococcus faecalis*, *Staphylococcus aureus*; and 1 type of fungus (*Saccharomyces cerevisiae*, 2%).

The HeLa&Zymo dataset contains 200ng of HeLa DNA and 200ng of ZymoBIOMICS Microbial Community DNA Standard computed by volume and listed concentrations. The sequenced samples were basecalled using Guppy v3.6.1 and aligned with reference downloaded from NCBI using Minimap2. We assigned the species of these sequences using the Minimap2 alignment because no species barcodes were used in this dataset. Due to the limited fungus sequences, only the bacterial sequences were kept for training, validation, and testing. The first 1500 signals for each read (about 150bp of sequence on average) were removed to avoid adapter sequences and signal instability.

The assembly of the Human&Zymo datasets each started with 400ng of Human GM12878 DNA and 400ng of ZymoBIOMICS HMW DNA Standard. They were barcoded using the SQK-RBK004 barcodes 1 and 2, 3 and 4, 5 and 6 respectively as indicated in the table. Then each dataset was generated using 200 ng of barcoded GM12878 (by volume) and 200ng of HMW Zymo (by volume) with a total of 400ng pooled in 10ul. For the Human&Zymo datasets, the extracted samples were basecalled using Guppy v3.6.1 with "–barcode_kits SQK-RBK004" specification to separate the reads and identify the barcodes. The barcodes were then used to identify the species of each read. The first 2000 (about 200bp worth of sequences on average) signals were removed to avoid barcode overfitting and signal instability. Based on the adapter, barcode and barcode flanking sequence description from the Nanopore Community [24, 25], 2000 signals should be more than enough to remove all above.

Each extracted signal read was normalized with fast5 scaling and offset. All reads were also normalized using Z-scored median absolute deviation. The extreme signal values with a modified z-score larger than 3.5 were replaced by the average of closest neighbors.

Model	Dataset	Layers	Channels	Window Size	Stride	Val Acc	Test Acc
VGG_1000	1M/20k/20k	2	[20]	[19, 5]	[6]	65.58	63.94
		2	[100]	[19, 5]	[6]	67.06	65.85
		4	[20, 50]	[19, 5, 6]	[3, 2]	70.93	69.33
		4	[40, 60, 100, 100]	[19, 5, 6]	[3, 2]	71.78	70.61
		6	[20, 50, 75]	[19, 5, 6, 3]	[3, 1, 1]	70.42	69.36
		6	[40, 75, 100]	[19, 5, 6, 3]	[3, 1, 1]	71.61	69.99
		8	[20, 20, 20, 20]	[20, 6, 6, 3, 3]	[2, 1, 1, 1]	72.21	71.16
		8	[30, 30, 30, 30]	[20, 6, 6, 3, 3]	[2, 1, 1, 1]	74.21	72.60
		8	[50, 50, 50, 50]	[20, 6, 6, 3, 3]	[2, 1, 1, 1]	76.63	75.99
		8	[20, 50, 75, 75]	[19, 5, 6, 3, 3]	[3, 1, 1, 1]	72.95	70.68
		10	[10, 20, 40, 80, 80]	[5, 5, 5, 5, 5]	[1, 1, 1, 1, 2]	77.46	76.73

Model	Dataset	Block	Layers	Val Acc	Test Acc
ResNet_1000	200k/10k/10k	BottleNeck	[2,2,2,2]	72.99	72.04
		BottleNeck	[1,1,1,1]	72.73	71.67
		BottleNeck	[1,1,1,1,1]	72.12	70.98
		BottleNeck	[2,2,1,1]	72.91	71.07
		BottleNeck	[1,1,2,2]	72.81	71.45
	1M/20k/20k	Basic	[2,2,2,2]	80.21	79.74
		BottleNeck	[2,2,2,2]	78.85	78.71
		+GroupChannel	[2,2,2,2]	77.95	77.56
+LSTM		BottleNeck	[2,2,2,2]	80.28	79.56
+AdamW		BottleNeck	[2,2,2,2]	79.90	79.88
Bonito_1000	1M/20k/20k	1*2	[8,8,16,16]	72.44	72.63
		2*2	[8,8,8,16,16]	73.49	74.36

Model	Dataset	Channels	Window Size	Hidden size	Layers	Bidirectional	Val Acc
CNN_3000+LSTM	1M/20k/20k	50	19	50	2	Yes	64.538
		75	19	75	4	Yes	64.238
CNN_3000+GRU		50	19	50	2	Yes	64.398
		75	19	75	4	Yes	64.692

Table S1: Selected Model Architecture Experiment Results and Hyperparameter Tuning Performance Report

Model Architecture Experiments and Hyperparameter Tuning

In this section, we describe some of the additional models and hyperparameter settings that we explored while developing SquiggleNet. Table S1 summarizes these results. Most of the model architectures were trained on the HeLa&Zymo dataset using 1000 signals per sequence, after removing the first 1500 signals. We used this shorter input length and a subset of the training dataset to enable more rapid model testing. We then re-trained some of the more promising models with 3000 signals per read. Each model was trained for 2 to 5 epochs until the validation accuracy started to plateau or show signs of overfitting.

The experiments based on the VGG_1000 architecture indicated that a model with more layers and more channels could generally offer better performance. However, the lack of shortcut skips in the architecture limited the possible depth of the VGG models.

We thus next experimented with a ResNet model with different numbers of layers, different numbers of blocks in each layer, different block types, and several other modifications. We found that increasing the number of blocks in each layer boosted performance somewhat, but increasing the number of layers did not necessarily improve the performance. Increasing the size of the training dataset boosted the model performance by ∼8% according to Table S1. We introduced a layer of LSTM in the middle of our ResNet model, and this increased performance by a small margin, but took much longer to train.

Recurrent Neural Network (RNN) models such as LSTM and GRU gave little advantage over CNN based models and also required significantly more resources to train. For the last set of experiments presented in Table S1, we used 3000 signals per read, introduced one layer of CNN with window size 19 to reduce the input size, and followed with various RNN architectures. The performance slowly increased from random guessing (50%), but plateaued around 65%. This could indicate that local features extracted by convolution provide sufficient information for classification, and long-range dependencies extracted by the recurrent network only help by a small amount.

Other models we tried include: a down-sized Bonito [26, 27], stacks of LSTM layers with variational window sizes, different hyperparameter settings, and different training datasets. After full consideration of model size, speed, performance, and training time, we settled on the best performing model architecture to perform the main experiments in the paper.

Comparison of Models Trained on HeLa&Zymo and Human&Zymo

In addition to the best-performing model trained on the HeLa&Zymo dataset, we conducted the same set of test experiments on a model trained on the Human&Zymo_b12 dataset. This dataset consists of a 1:1 mix of human DNA and Zymo HMW DNA, as described in Supplement Section Data Collection. We tested on all five datasets, which include different sample preparations, flow cells, sample components, and human:bacteria ratios. The performance can be found in Figure S1. The overall performance is almost as well as Figure 2. This may be because the Human&Zymo_b12 dataset contains less training data than the HeLa&Zymo dataset. Overall, this analysis indicates that the model achieves high accuracy

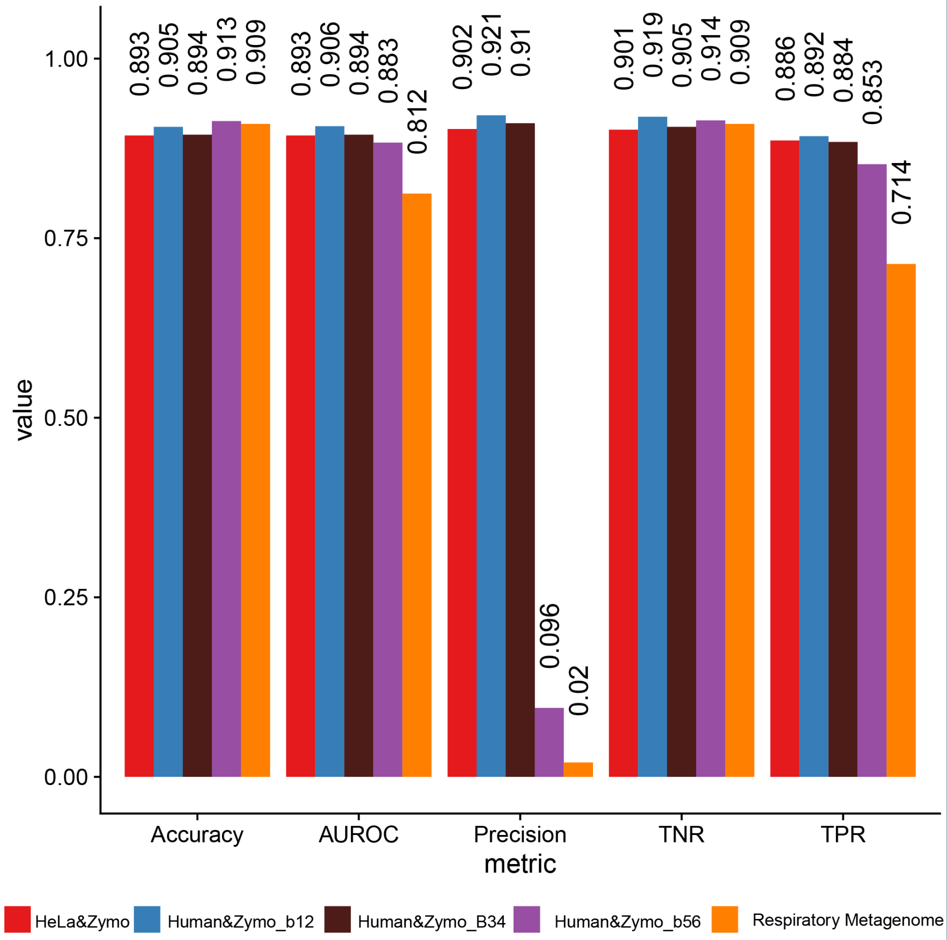

Figure S1: **Overall Performance across five test Datasets with Model trained on Human&Zymo_b12:** Accuracy, True Positive Rate (TPR, RECALL), True Negative Rate (TNR), Precision, and the AUROC score of the model trained on the HeLa&Zymo training set, and tested on five test sets with bacterial sequences as the target.

whether trained on HeLa DNA (which is a highly mutated cancer cell line) or GM12878 DNA (which should look more like healthy human DNA), when tested on the other type of data.

Comparison Experiment Method Details

The performance and efficiency comparison experiment was conducted on dataset Human&Zymo_b34 with 1:4 Human and Zymo mix. For a fair read-in and write-out time comparison across the platforms, all the reads were pre-truncated into 3000 signals, 6000 signals, and full length, after removing the first 2000 signals for adapters and barcode. Any reads shorter than the minimum input requirements were discarded. The ground truth labels for each read were obtained by the SQK-RBK004 barcode 3 and 4, basecalled by Guppy v3.6.1. Any reads that were labeled as other barcodes or not labeled were discarded.

SquiggleNet was tested on 1) a single-usage Intel(R) Xeon(R) CPU E5-2697v3 @ 2.60GHz machine with a single TITAN Xp GPU with batch size one (fast5 file, about 4000 sequences each), and 2) a single-usage 3.5 GHz Dual-Core Intel Core i7 Macbook Pro with a single thread. Over 90% of the compute time on GPU was spent on file read-in and write-out (over 40% of the time on CPU).

When testing the Guppy+Minimap2 method, we used 4 callers and 4 runners per GPU for Guppy, and 32 threads for Minimap2 sequence alignment. Indexes for both human and Zymo community reference genome were pre-generated. The test datasets with 3000 signals per read, 6000 signals per read, and full length reads were basecalled by Guppy and then aligned using Minimap2 with the pre-generated index. The resulting read IDs were cross-referenced with the ground truth labels for barcode 3 and 4 to calculate the overall accuracy.

When testing the UNCALLED method, we used 32 threads to process 3000 signals, 6000 signals, and full-length reads. BWA index was pre-generated for the Zymo community reference genome. Unfortunately, UNCALLED struggles to map repetitive references longer than 100Mbp, such as the human genome. Therefore, we had to treat all the reads that weren't classified as Zymo to be human reads as an approximation for the performance computation. The resulting read IDs were cross referenced with the ground truth labels for barcode 3 and 4, to calculate the overall accuracy.

DNA Methylation Differences Between Human and Bacterial DNA

	Avg mAs/KB	Avg mCs/KB
Bacterial	1.72	3.53
Human	0.01	16.27

Table S2: Average number of methylated A (mA) and methylated C (mC) nucleotides in the first 1000 nucleotides of human and bacterial DNA sequences

Since SquiggleNet achieves higher classification accuracy than base calling followed by sequence alignment (given 3000 signals), we hypothesized that the neural network may be using other information besides just nucleotide sequence. One possible such source of information is methylation. We therefore investigated whether there are systematic methylation differences between the human and bacterial DNA sequences.

To do this, we used Guppy v3.6.1 to output the methylation probability for each sequence in Dataset Human&Zymo_b34. For each base pair with greater than zero percent chance to be an A or C (the only nucleotides for which Guppy will predict methylation), we computed the most likely base call: A, methylated A, C, or methylated C. We then calculated the total and average numbers of methylated As and methylated Cs in the first 1000 bases of both human and bacterial sequences.

As shown in Table S2, bacterial sequences contain on average about 2 methylated A nucleotides and 4 methylated C nucleotides per kilobase, whereas the human sequences contain on average 0 methylated As and 16 methylated Cs per kilobase. This suggests that methylation is indeed a possible feature that could help to classify sequences as bacterial or human.

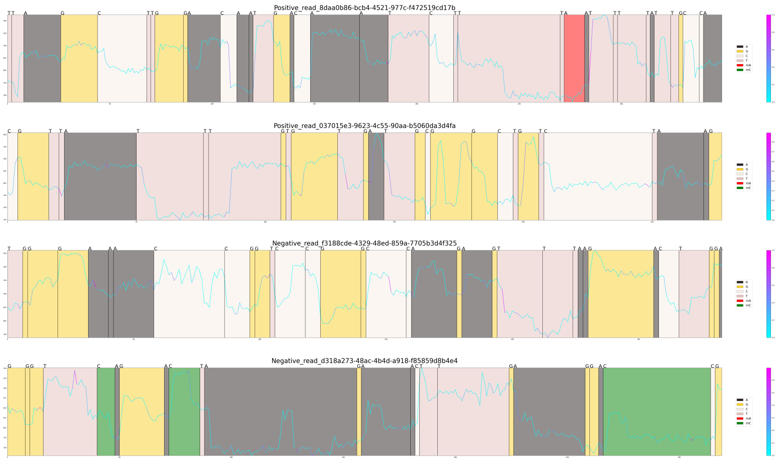

Figure S2: **SquiggleNet Integrated Gradients and Signal Dynamics** Four sample signal chunks, 2 positive signals (top), and 2 negative signals (bottom). Each signal chunk was colored with corresponding predicted nucleotide (from Guppy basecalling results). Methylated As were colored in red, and methylated Cs were colored in green. Each squiggle was also colored by the value of the integrated gradient. A higher integrated gradient value (purple) indicates that the model is more excited by that part of the signal, and thus that it contributes more to the final classification decision.

Model Interpretation Using Integrated Gradients

We used the method of integrated gradients (IG) [15] to investigate the relationship between convolutional filters, sequencing signal dynamics, and classification results from SquiggleNet. Integrated gradients computes the amount of gradient change for each corresponding input, and by doing so, offers interpretation on which part of the input contributes the most to the model's decision. The IG approach gives a way of attributing model outputs to specific input features. In our case, IG can tell us which electrical signal values over the course of the sequencing time most strongly influence SquiggleNet's decision to classify the sequence as positive or negative.

To investigate this, we adapted an existing PyTorch implementation [28] of IG so that it works for 1-D squiggles, rather than 2-D images. We used a squiggle whose value is identically the mean pore conductance (which is identically 0 after normalization) as the background signal required by IG. This is analogous to using an all-black image (the standard background signal for performing IG on image data). Then we simultaneously plotted the raw squiggle, base calls, and integrated gradients . Figure S2 shows the results for two positive and two negative sequences, with one positive and one negative containing a methylated nucleotide. The attribution is clearly strongest at positions where the signal changes direction and/or changes by a large amount. Attribution also tends to be high at the nucleotide boundaries predicted by the base caller. This suggests that SquiggleNet has learned filters related to the nucleotide composition of the signal and uses the results to make classification decisions.

Throughput Estimation Model

The throughput estimation model computes the total amount of time t and the number of base pairs l needed to achieve x targeted reads, and compares these values with and without read-until.

The estimation model assumes the average targeted read length to be $\bar{z}$, and the average non-targeted read length to be $\bar{h}$. It assumes the total number of functioning pores k is constant throughout the sequencing run. It also assumes all the functioning pores are actively sequencing or in the process of getting a new read. All the other parameter values can be found in table S3.

Table S3: Throughput Estimation Model Parameters

Description	Symbol	Value
Total sequencing time	t	
Total base pair sequenced	l	
Number of targeted reads	x	1000
Number of total reads	$n = x/a$	
Number of pores alive	k	400
Average sequencing speed	v	450 bp/s
Average read length: target	$\bar{z}$	40000 bp
Average read length: non-target	$\bar{h}$	40000 bp
Concentration: target	a	0.1
Concentration: non-target	$b = 1 - a$	0.9
Classifier TPR	p_1	90%
Classifier TNR	p_2	90%
Time: attract a new read	t_0	0.01s
Time: initial sequencing	t_1	1s
Time: decision time	t_2	0.8s
Time: reject a read	t_3	0.01s

Without Read-Until, the total sequencing time t_{wo} and the total number of base pairs l_{wo} needed for x targeted sequences are:

$$l_{wo} = [\bar{z}a + \bar{h}b]n$$

$$t_{wo} = [(t_0 + \bar{z}/v)a + (t_0 + \bar{h}/v)b] \cdot n/k$$

With Read-Until, the total sequencing time t_{ru} and the total number of base pairs l_{ru} needed for x targeted sequences are:

$$l_{ru} = [(t_1 v + t_2 v)((1 - p_1)a + bp_2) + \bar{z}ap_1 + \bar{h}b(1 - p_2)]n$$

$$t_{ru} = [(t_0+t_1+t_2+t_3)bp_2+(t_0+t_1+t_2+t_3)a(1-p_1)+(t_0+\bar{z}/v)ap_1+(t_0+\bar{h}/v)b(1-p_2)]\cdot n/k$$

Several parameters cancel out and thus do not affect the sequencing length ratio l_{wo}/l_{ru} or the sequencing time ratio t_{wo}/t_{ru}. These parameters include: the number of targeted reads x, the number of total reads n, and the number of active nanopores k. Several hyperparameters were set to default values based on SquiggleNet's statistics and the sample means from multiple sequencing experiments. These include average sequencing speed ($v = 450$ bp/s), classification TPR ($p_1 = 90\%$), classification TNR ($p_2 = 90\%$), initial sequencing time ($t_1 = 1s$), and decision time ($t_1 = 0.8$). The average read length for target and non-target reads are both set to 40000 bp. We set the target concentration to 10% and non-target read concentration to 90%.

We also investigated how t_0, the time to attract a new read, or t_3, affect the throughput ratio. We varied these two parameters from 0 to 1 second and plotted the resulting throughput ratio for both sequencing length and sequencing time (Figure S3). Neither parameter significantly affected the throughput ratio.

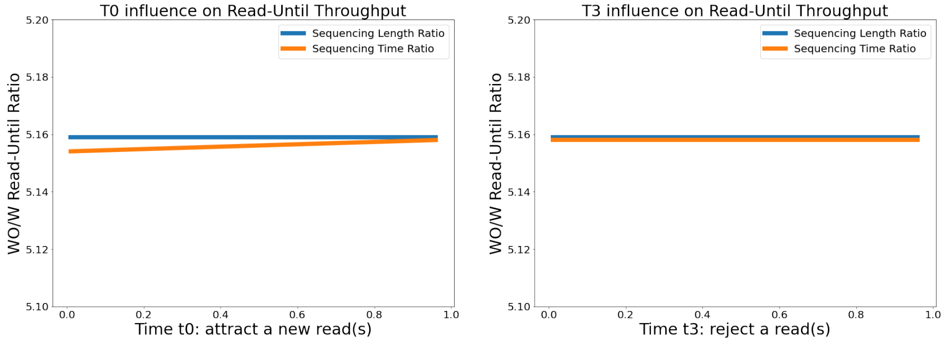

Figure S3: **The influence of t0 and t3 on Read-Until Throughput:** As t0 and t3 change from 0 to 1 second, the sequencing time and length ratio (without/with read-until) are not effected significantly.

Model Performance on Different Test Dataset Composition Ratio

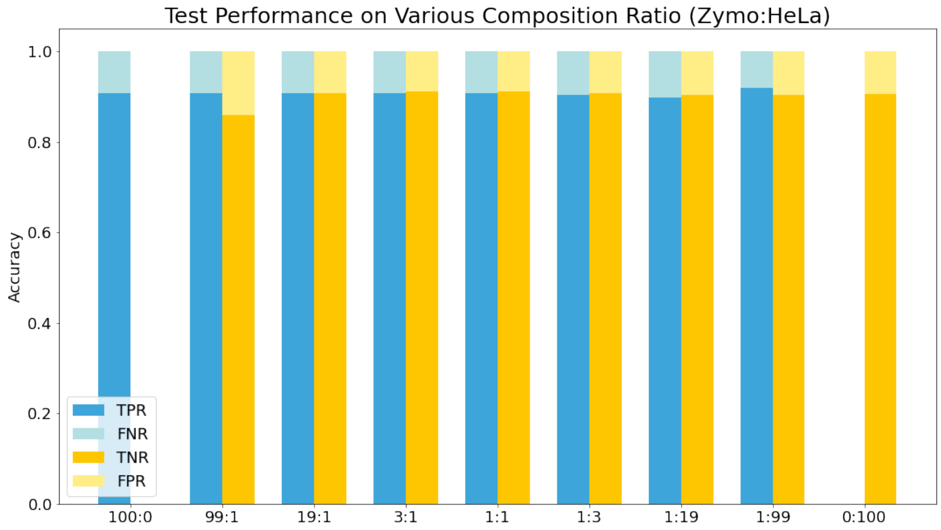

Figure S4: **Robustness of SquiggleNet Performance Across Varying Test Set Composition:** The TPR, FNR, TNR, and FPR are independent of the test dataset Zymo:HeLa ratio and remain around 90%.

Because the trained model makes predictions independently for each individual sequence, the true positive, false positive, false negative, and true negative rates are independent of the distribution of the test set. In contrast, the ratio of sequences in the training dataset is important, which is why we conducted training with equal numbers of positive and negative sequences. To confirm this, we used the model

pretrained on the HeLa&Zymo dataset (same as reported in Figure 2), and tested it on datasets with HeLa&Zymo composition ratios ranging from 100:0, 99:1, 19:1, 3:1, 1:1, to 1:3, 1:19, 1:99 to 0:100. As Figure S4 shows, the TPR/TNR/FNR/FPR values are not significantly affected by the test composition ratio and remain around 90%. The absolute number of positive and negative predictions will of course vary with the composition of the test dataset, but this behavior is desirable.

Model Performance on Odd and Even HeLa Chromosomes

We trained a new model on the HeLa & Zymo dataset using even chromosomes for training, and odd for testing, each with a 1:1 ratio of HeLa and Zymo sequences. We omitted all chromosomes with non-numeric names–including the mitochondrial chromosome, sex chromosomes, and other contigs–rather than arbitrarily designating them as odd or even. Otherwise we followed the training procedure exactly as described in the Methods. The analysis indicates that SquiggleNet can correctly classify reads from missing chromosomes (Table S4). The performance is about 6% higher than what we obtained using all chromosomes. This is possibly due to the chromosomes we excluded, which may be enriched for low-quality sequences. In addition, the human mitochondrial chromosome is somewhat phylogenetically similar to prokaryotic DNA, so excluding it may have also boosted the performance.

Accuracy	Precision	TPR (Recall)	TNR
96.84%	96.97%	96.65%	97.02%

Table S4: Model Performance on Odd and Even HeLa Chromosomes
