# Supplementary material for: SquiggleNet: real-time, direct classification of nanopore signals
Source: Genome Biol. 2021 Oct 27;22:298. doi: 10.1186/s13059-021-02511-y (PMC8548853; doi:10.1186/s13059-021-02511-y)
Supplement: Supplementary file 2 — Additional file 2 Review history. [file 13059_2021_2511_MOESM2_ESM.docx]

Review History

**First round of review**

**Reviewer 1**

**Were you able to assess all statistics in the manuscript, including the appropriateness of statistical tests used?**

No

**Were you able to directly test the methods?**

No

**Comments to author:**

The authors present SquiggleNet, a highly efficient tool for real-time identification of nanopore reads while molecules are passing through the pore. Advances in this space are very promising, and the presented implementation seems to bypass some of the limitations of existing approaches for selective sequencing. I cannot comment on specific deep learning implementation details as that goes beyond my expertise. The manuscript is clear and well written.

Please find my specific comments below.

Sincerely,

Wouter De Coster

1) It is interesting to see that SquiggleNet uses local sequence context/nucleotide composition or nucleotide modifications for its decision, which makes me wonder how robust it could be in the case of missing human training data. For example, would training on just the odd-numbered human chromosomes (and bacterial reads) still enable the correct classification of reads derived from the even-numbered human chromosomes?

2) The leave-one-out experiment is similarly very interesting and well-executed, but I am a bit confused about the conclusion. I expect that what is meant is that when leaving out one bacterial species SquiggleNet can still identify reads thereof as "bacterial" without, of course, being able to label those as "the missing species"? If my understanding is correct, I would suggest revising the final sentence (page 8, lines 23-26) to "In summary, these two sets of experiments show that even when one bacterial species was not seen during training time, SquiggleNet was still able to identify its reads as bacterial with high confidence." or similar.

3) It is unclear to me if the current SquiggleNet implementation is something that users can start using immediately in their experiments for selective sequencing, or if further changes have to be made to enable the software to interact with the MinKNOW API. The GitHub Readme suggests the latter.

4) I would suggest the authors clarify earlier in their manuscript that their selective sequencing approach is currently only for selection on the species level, rather than on the locus level, an application that is enabled with UNCALLED and readfish (e.g. selectively sequencing specific human genes). While the discussion mentions this extension to selective sequencing of human loci would be a possibility it is unclear to me how this would be achieved. Would the local sequence information be sufficiently different from one gene or a set of genes to the rest of the genome?

5) And as a minor comment: the authors mention that the longest nanopore read is around 2Mbp, while I believe longer reads up to 4Mb have been reported but not formally published beyond Twitter.

**Reviewer 2**

**Were you able to assess all statistics in the manuscript, including the appropriateness of statistical tests used?**

**No**

**Were you able to directly test the methods?**

No

**Comments to author:**

I have read through the manuscript entitled "Real-Time, Direct Classification of Nanopore Signals with SquiggleNet" by Bao et al. In the manuscript, the authors presented a deep-learning based method for classifying DNA sequences directly from electrical signals, which is interesting and nontrivial for the current growth of nanopore sequencing data involved in genomics. Herein the authors proposed a challenge to distinguish human DNA from bacterial DNA just using their partial information of electronic signals, the advantage of their work seemed to be base-pair sequence-free, and be able to evidently save sequencing time and cost. I believe that the work would be helpful for a given task during the nanopore sequencing processes. Herein, I would like to present my comments as follows.

Major comments:

(1) What I have a serious concern, is what a kind of situation, or task setting, proposed by the authors in their work. The authors declared that "SquiggleNet distinguished human from bacterial DNA", while when the authors tested SquiggleNet they seemed to just evaluate the performance of distinguishing bacterial DNA reads from human reads. Perhaps the two operations makes little difference when two kinds of reads (human and bacterial) were similar in reads number, but significant difference should be noted when the opposite happens, especially the reads number of human DNA much less than number of bacterial DNA reads. In this case the evaluation metric, especially the false prediction ratio, should be reported. Therefore, the authors should clarify the ratio of number of human DNA reads and bacteria reads for the test set, and should also address the real situation of distinguishing ones with small number of reads from the other with large number of reads. The analysis for respiratory metagenomic data perhaps was perhaps able to show the reasonable evaluation on real situation, for example the false positive and negative prediction ratio.

(2) I have also a concern that the bacterial stains used in training set as well as in test set have overlap, maybe I did not understand the authors' description in the manuscript. However, I note that the first set, ZymoBIOMICS Microbial Community DNA Standard (8 bacteria genomes were selected in this manuscript: Pseudomonas aeruginosa, Escherichia coli, Salmonella enterica, Lactobacillus fermentum, Enterococcus faecalis, Staphylococcus aureus, Listeria monocytogenes, Bacillus subtilis), and the other three sets were simulated based on 7 bacteria genoems (Pseudomonas aeruginosa, Escherichia coli, Salmonella enterica, Enterococcus faecalis, Staphylococcus aureus, Listeria monocytogenes, Bacillus subtilis, listed by ZymoBIOMICS HMW DNA Standard). Several genomes are identical, while some of them are closely related in phylogenetics. So, the authors should clarify how to handle the overlapped information of these bacterial strains.

Major comments:

(1) Figure 3b and 4, the Y axis, "Accuracy" should be added with "(%)". Also, the digits after the decimal point should also be consistent.

(2) Line 61-62, Page 5："the target to non-target sequence ratio is 99 to 1", herein "99 to 1" seems to be "1 to 99"?

**Authors Response**

**Point-by-point responses to the reviewers’ comments:**

Reviewer 1

The authors present SquiggleNet, a highly efficient tool for real-time identification of nanopore reads while molecules are passing through the pore. Advances in this space are very promising, and the presented implementation seems to bypass some of the limitations of existing approaches for selective sequencing. I cannot comment on specific deep learning implementation details as that goes beyond my expertise. The manuscript is clear and well written.

Please find my specific comments below.

Sincerely,

Wouter De Coster

1) It is interesting to see that SquiggleNet uses local sequence context/nucleotide composition or nucleotide modifications for its decision, which makes me wonder how robust it could be in the case of missing human training data. For example, would training on just the odd-numbered human chromosomes (and bacterial reads) still enable the correct classification of reads derived from the even-numbered human chromosomes?

*We trained a new model on the HeLa & Zymo dataset using even chromosomes for training, and odd for testing, each with a 1:1 ratio of HeLa and Zymo sequences. We omitted all chromosomes with non-numeric names--including the mitochondrial chromosome, sex chromosomes, and other contigs--rather than arbitrarily designating them as odd or even. Otherwise we followed the training procedure exactly as described in the Methods. We added the results to a supplementary table (reproduced below). The analysis indicates that SquiggleNet can, in fact, correctly classify reads from missing chromosomes. The performance is actually about 6% higher than what we reported in the main paper. This is possibly due to the mitochondrial and other chromosomes we excluded, which may be enriched for low-quality sequences.*

2) The leave-one-out experiment is similarly very interesting and well-executed, but I am a bit confused about the conclusion. I expect that what is meant is that when leaving out one bacterial species SquiggleNet can still identify reads thereof as "bacterial" without, of course, being able to label those as "the missing species"? If my understanding is correct, I would suggest revising the final sentence (page 8, lines 23-26) to "In summary, these two sets of experiments show that even when one bacterial species was not seen during training time, SquiggleNet was still able to identify its reads as bacterial with high confidence." or similar.

*Your interpretation is correct. We can identify reads from the missing species as bacterial rather than human, but our model does not distinguish among bacterial species. We did not investigate whether the model can classify a read as Salmonella vs. E. coli, for example. We have updated the text to clarify this point.*

3) It is unclear to me if the current SquiggleNet implementation is something that users can start using immediately in their experiments for selective sequencing, or if further changes have to be made to enable the software to interact with the MinKNOW API. The GitHub Readme suggests the latter.

*The current SquiggleNet implementation takes raw fast5 files as input and outputs prediction results (0 and 1) for each read. Essentially the only piece needed for integration with read-until is writing a few lines of code to check the classification result and eject the reads classified as non-target. We have updated the GitHub Readme to clarify this point.*

4) I would suggest the authors clarify earlier in their manuscript that their selective sequencing approach is currently only for selection on the species level, rather than on the locus level, an application that is enabled with UNCALLED and readfish (e.g. selectively sequencing specific human genes). While the discussion mentions this extension to selective sequencing of human loci would be a possibility it is unclear to me how this would be achieved. Would the local sequence information be sufficiently different from one gene or a set of genes to the rest of the genome?

*Thank you for this interesting and helpful suggestion. To investigate whether our approach works for selective sequencing on the locus level, we trained SquiggleNet on a published dataset from Cas9-directed enrichment of human interspersed repeat elements (McDonald et al. Nature Communications 2021). We trained the model to classify reads as repeat-containing or non-repeat-containing. The dataset is significantly smaller than what we previously used for the species classification (170k positive reads and 170k negative reads, compared to 1.2M each for the HeLa & Zymo dataset). However, we still obtained accuracy > 92%. These results demonstrate that our model is indeed able to accurately distinguish a set of loci within the human genome. The accuracy would likely improve further if more training data were available. We added a results subsection and figure (Fig. 7, reproduced below) summarizing these results.*

*Figure 7. Identifying Reads Containing Interspersed Repeat Elements. (a) Diagram of experimental strategy for enriching human mobile elements, including interspersed repeats. A guide RNA specific to each repeat class directs Cas9 to cut the DNA and ligate a sequencing adapter. However, adapters are also ligated to some sequences without repeat elements. Subsequent nanopore sequencing produces both target and non-target reads. (b) Pie charts of the proportion of L1H repeat elements from Cas9 enrichment only vs. SquiggleNet classification. (c) Classification metrics demonstrating SquiggleNet's ability to distinguish reads with or without L1H repeats.*

5) And as a minor comment: the authors mention that the longest nanopore read is around 2Mbp, while I believe longer reads up to 4Mb have been reported but not formally published beyond Twitter.

*We have updated the text to indicate that the longest published example is 2MB, although longer reads have been reported anecdotally.*

Reviewer 2

I have read through the manuscript entitled "Real-Time, Direct Classification of Nanopore Signals with SquiggleNet" by Bao et al. In the manuscript, the authors presented a deep-learning based method for classifying DNA sequences directly from electrical signals, which is interesting and nontrivial for the current growth of nanopore sequencing data involved in genomics. Herein the authors proposed a challenge to distinguish human DNA from bacterial DNA just using their partial information of electronic signals, the advantage of their work seemed to be base-pair sequence-free, and be able to evidently save sequencing time and cost. I believe that the work would be helpful for a given task during the nanopore sequencing processes. Herein, I would like to present my comments as follows.

Major comments:

(1) What I have a serious concern, is what a kind of situation, or task setting, proposed by the authors in their work. The authors declared that "SquiggleNet distinguished human from bacterial DNA", while when the authors tested SquiggleNet they seemed to just evaluate the performance of distinguishing bacterial DNA reads from human reads. Perhaps the two operations makes little difference when two kinds of reads (human and bacterial) were similar in reads number, but significant difference should be noted when the opposite happens, especially the reads number of human DNA much less than number of bacterial DNA reads. In this case the evaluation metric, especially the false prediction ratio, should be reported. Therefore, the authors should clarify the ratio of number of human DNA reads and bacteria reads for the test set, and should also address the real situation of distinguishing ones with small number of reads from the other with large number of reads. The analysis for respiratory metagenomic data perhaps was perhaps able to show the reasonable evaluation on real situation, for example the false positive and negative prediction ratio.

*Because the trained model makes predictions independently for each individual sequence, the true positive, false positive, false negative, and true negative rates are independent of the distribution of the test set. In contrast, the ratio of sequences in the training dataset is important, which is why we conducted training with equal numbers of positive and negative sequences. We reported the results from test datasets containing varying ratios of human and bacterial reads in Table 2. The true positive rate (bacteria as target) and the true negative rate were reported in Figure 2 and Figure 8, and are around 90% in most of the reported cases. The false positive and false negative rates are therefore under 10% correspondingly.*

*To further address your concern, we conducted additional experiments using the model trained on the Hela & Zymo dataset and tested on different HeLa & Zymo composition ratios (Fig. 12, reproduced below). These analyses confirmed that the TPR, TNR, FNR, and FPR are not significantly affected by the species ratio in the test dataset, and the accuracies remain around 90%. The absolute number of positive and negative predictions will of course vary with the composition of the test dataset, but this behavior is desirable.*

*Figure 12: Robustness of SquiggleNet Performance Across Varying Test Set Composition. The TPR, FNR, TNR, and FPR are independent of the test dataset Zymo:HeLa ratio and remain around 90%.*

(2) I have also a concern that the bacterial stains used in training set as well as in test set have overlap, maybe I did not understand the authors' description in the manuscript. However, I note that the first set, ZymoBIOMICS Microbial Community DNA Standard (8 bacteria genomes were selected in this manuscript: Pseudomonas aeruginosa, Escherichia coli, Salmonella enterica, Lactobacillus fermentum, Enterococcus faecalis, Staphylococcus aureus, Listeria monocytogenes, Bacillus subtilis), and the other three sets were simulated based on 7 bacteria genoems (Pseudomonas aeruginosa, Escherichia coli, Salmonella enterica, Enterococcus faecalis, Staphylococcus aureus, Listeria monocytogenes, Bacillus subtilis, listed by ZymoBIOMICS HMW DNA Standard). Several genomes are identical, while some of them are closely related in phylogenetics. So, the authors should clarify how to handle the overlapped information of these bacterial strains.

*Although some of the species do indeed overlap between some of our training and testing datasets, this does not compromise our conclusions about the usefulness and generalizability of our approach. In particular, we were careful to evaluate the performance of our method to confirm that it generalizes to unseen species. We chose to use the ZymoBIOMICS Microbial Community DNA Standard and the closely related ZymoBIOMICS HMW DNA Standard because these are widely used benchmarks for microbial sequencing applications. An additional benefit is that these mixtures contain both similar and distantly related bacterial species, allowing us to evaluate how well the model generalizes.*

*The usefulness of our model comes from its ability to be trained on individual reads from a dataset within one domain, and tested on different individual reads from a new dataset within the same domain. Some overlap in the species between the training and testing datasets is necessary for the model to learn the characteristics of the task, and perform predictions on a completely different test dataset within the same domain.*

*In our experiments (Fig. 2, for example), we split the HeLa & Zymo dataset into training, validation, and testing (Table 2) datasets. These reads came from the same organisms (HeLa and 8 bacteria species in the Zymo community), but the individual reads do not overlap between training and testing datasets. Our model is able to learn the inherent features of each species during training, and perform high-accuracy predictions during testing.*

*Moreover, we tested the same model on the other three datasets: Human&Zymo_b12, Human&Zymo_b34, Human&Zymo_b56. Compared to the training dataset, there’s an overlap on the genomic information (human vs. HeLa, 8 bacterial species vs. 7), meaning the model is expected to perform well with trained weights, and it did achieve over 90% in accuracy. However, these new datasets were also from different nanopore sequencing runs, with different sample preparations, which yields significant technical variability, and our model was still able to extract the key features from the raw sequencing signals to distinguish the species of each individual read.*

*We further evaluated the model performance on test datasets containing individual reads from bacterial species not seen during training. To do this, we systematically held out training reads from one species at a time and evaluated the model’s ability to classify reads from the held-out species as bacteria rather than human (Fig. 4). We tested the same model (trained on the HeLa & Zymo dataset) on a Respiratory Metagenome dataset (Table 2 and Fig. 5) which overlaps less than 3% with the bacterial species in the training dataset. Our model still performed over 90% in accuracy and over 72% in the true positive rate. This result demonstrated the model’s robustness in learning the inherent difference between the human genome and a broad range of bacterial species.*

*In summary, SquiggleNet works well at classifying individual reads as bacterial or human when the reads come from species seen during training and even when the reads come from unseen bacterial species.*

Major comments:

(1) Figure 3b and 4, the Y axis, "Accuracy" should be added with "(%)". Also, the digits after the decimal point should also be consistent.

(2) Line 61-62, Page 5："the target to non-target sequence ratio is 99 to 1", herein "99 to 1" seems to be "1 to 99"?

*We have corrected these issues.*

**Second round of review**

**Reviewer 1**

Dear authors,

I am satisfied by the answers to the queries I raised during my previous review, and have no further comments. I wish to congratulate the authors on their outstanding work.

Sincerely,

Wouter De Coster
